# Functional Differences Exist between TNFα Promoters Encoding the Common −237G SNP and the Rarer HLA-B*5701-Linked A Variant

**DOI:** 10.1371/journal.pone.0040100

**Published:** 2012-07-13

**Authors:** Peter D. Simpson, Eirini Moysi, Kate Wicks, Kritika Sudan, Sarah L. Rowland-Jones, Andrew J. McMichael, Julian Knight, Geraldine M. Gillespie

**Affiliations:** 1 MRC Human Immunology Unit, Weatherall Institute of Molecular Medicine, John Radcliffe Hospital University of Oxford, Oxford, Oxfordshire, United Kingdom; 2 Wellcome Trust Centre for Human Genetics, University of Oxford, Oxford, Oxfordshire, United Kingdom; University of London, St George's, United Kingdom

## Abstract

A large body of functional and epidemiological evidence have previously illustrated the impact of specific MHC class I subtypes on clinical outcome during HIV-1 infection, and these observations have recently been re-iterated in genome wide association studies (GWAS). Yet because of the complexities surrounding GWAS-based approaches and the lack of knowledge relating to the identity of rarer single nucleotide polymorphism (SNP) variants, it has proved difficult to discover independent causal variants associated with favourable immune control. This is especially true of the candidate variants within the HLA region where many of the recently proposed disease influencing SNPs appear to reflect linkage with ‘protective’ MHC class I alleles. Yet causal MHC-linked SNPs may exist but remain overlooked owing to the complexities associated with their identification. Here we focus on the ancestral TNFα promoter −237A variant (rs361525), shown historically to be in complete linkage disequilibrium with the ‘protective’ HLA-B*5701 allele. Many of the ancestral SNPs within the extended TNFα promoter have been associated with both autoimmune conditions and disease outcomes, however, the direct role of these variants on TNFα expression remains controversial. Yet, because of the important role played by TNFα in HIV-1 infection, and given the proximity of the −237 SNP to the core promoter, its location within a putative repressor region previously characterized in mice, and its disruption of a methylation-susceptible CpG dinucleotide motif, we chose to carefully evaluate its impact on TNFα production. Using a variety of approaches we now demonstrate that carriage of the A SNP is associated with lower TNFα production, via a mechanism not readily explained by promoter methylation nor the binding of transcription factors or repressors. We propose that the −237A variant could represent a minor causal SNP that additionally contributes to the HLA-B*5701-mediated ‘protective’ effect during HIV-1 infection.

## Introduction

As demonstrated by both epidemiological findings and in functional studies the MHC locus represents a crucial host factor that strongly impacts the clinical course of HIV-1 infection [1], [2]. The importance of the MHC region has also been re-affirmed in recent genome wide association (GWAS) studies in both Caucasian and African-American patient cohorts [3], [4], [5], [6], [7]. It remains unknown, at present, if specific single nucleotide polymorphisms (SNPs) within this region constitute additional variables that independently or synergistically associate with viral control. Although potential candidates have been identified, most of these are assumed to reflect linkage with MHC class I types known to associate with a slower course of disease progression. Nonetheless, causal SNPs in this region may exist but remain overlooked owing to the method of SNP-tagging which underlies GWAS approaches, and also due to the lack of knowledge relating to the identity of rare SNP variants.

Given the strong association of HLA-B*5701 with sustained viral control and prolonged AIDS-free survival, the impact of SNPs in strong linkage with this allele warrants further investigation. Of note are TNFα promoter SNPs, which are in linkage disequilibrium (LD) with a number of ancient HLA haplotypes, and have been implicated both positively and negatively with disease outcomes in the setting of various viral/bacterial infections and autoimmune conditions. Of particular interest is the TNFα promoter −237G to A SNP (rs361525) which is in complete LD with the ‘protective’ MHC class I HLA-B*5701 subtype. This SNP maps to the ancestral 57.1 haplotype (characterised historically by the presence of HLA-A*01, HLA-C*06, DRB1*07, DQB1*0303 and various *Bf, C4A, C4B,* and TNFα/*β* related-polymorphisms), and is also linked to HLA-B*5701 in the absence of HLA haplotype-defining genes [8]. The −237 SNP is located close to the proximal promoter, within a CpG dinucleotide motif, and maps to a region corresponding to a putative repressor site previously identified in mice [9].

TNFα is an important pleiotropic pro-inflammatory cytokine, which plays a fundamental role in both innate and adaptive immunity. Yet excessive TNFα is associated with various autoimmune, inflammatory and infectious pathologies, hence its production is tightly regulated *in vivo*. Transcription of TNFα is controlled by a highly conserved 200 bp core promoter, which acts as a nucleation site for the binding of a diverse array of transcription factors (Tfs) and co-activators; together, these form a multi-component nucleosome (the TNFα enhanceosome) of which the exact composition is dictated by the nature of the inducing stimulus [10]. The distal TNFα promoter extends some 1.5 kB upstream of the Transcription Start Site (TSS) and includes the ancient HLA-associated SNP variants - although this region is not directly required for promoter activity it can modulate TNFα production [11]. Consistent with this is the identification of Tf binding sites within the extended promoter region, of which a number encompass the ancestral HLA-linked SNPs, highlighting their potential to affect TNFα production *in vivo* [12], [13], [14], [15]. However, the precise functional impact of these SNPs remains controversial, and this includes the HLA-B*5701-linked −237A SNP. The relevance of this promoter polymorphism from the viewpoint of HIV-1 infection has also not been carefully evaluated. This is particularly relevant in light of the role played by this cytokine in relation to HIV-1 replication, viral spread and generalized immune activation. In this study we evaluate the significance of the −237A SNP, and investigate its functional impact in relation to the common −237G variant.

## Results

### Analysis of −237 SNP Variant Promoters using a Luciferase Reporter Assay System

**Figure 1:**
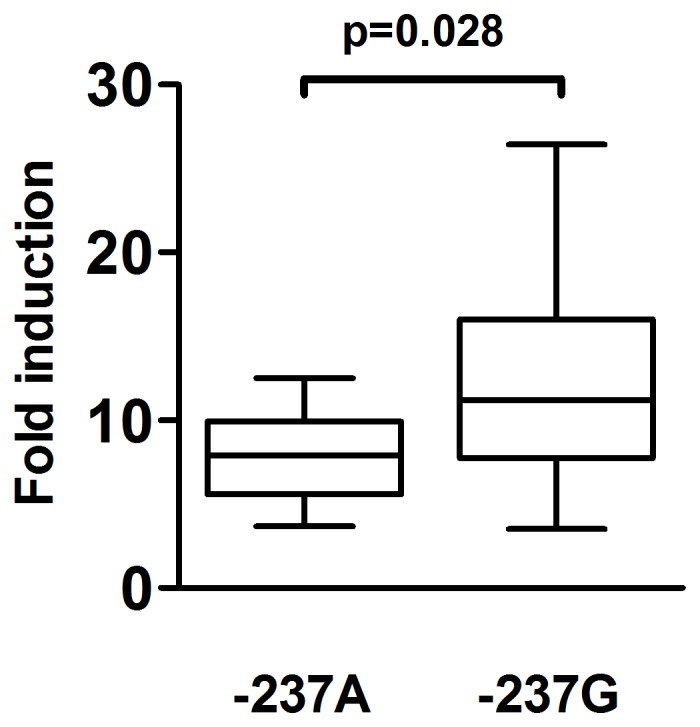
TNFα promoters encoding the −237A SNP display reduced activity following stimulation in a luciferase reporter assay system. Jurkat cells were transfected with reporter luciferase plasmids under the control of TNFα promoters carrying either the −237A or G variant. Following 24 hours, transfected cells were stimulated for 4 hours with PMA/ionomycin or left untreated following which reporter gene activity was measured. The data is presented as fold change (in relative light units (RLU)), and represents differences in RLU between stimulated and non-stimulated cells for each of the transfected variants. The cat whisker plots illustrate pooled results obtained from 4 independent transfection assays, where each transfection included 5 replicas, and denotes median luciferase induction, standard deviation, the upper and lower quartiles and the data range. Wilcoxon-Mann-Whitney tests were used for statistical comparisons, and two-tailed P values are indicated.

Firstly, we questioned if the single G to A polymorphism at position −237 directly affected the activity of the TNFα promoter, and using a luciferase reporter system we evaluated the impact of both promoter polymorphisms on luciferase gene expression. This approach was chosen to remove any potential interference from post-transcriptional control mechanisms targeting the TNFα mRNA 3′ UTR region [16]. Jurkat E6.1 cells transfected with promoter-reporter luciferase plasmids carrying the variant −237 SNPs were tested for both basal and stimulated (PMA/ionomycin) luciferase activity. Although we performed dual luciferase assays to normalise for differences in transfection efficiencies, the thymidine kinase control vector (pRL-TK) used for data normalization was adversely affected by PMA/ionomycin stimulation in Jurkat cells as reported elsewhere [17], [18]. Hence we only compared fold induction for the individual promoters (stimulated/basal) and not relative differences in the absolute luciferase activity for the variant promoter transfections following activation. Collectively, our results demonstrated that the −237A SNP resulted in weaker luciferase induction relative to promoters encoding the −237G SNP upon stimulation. This result was consistently observed in follow-up experiments using new preparation of plasmids and fresh cultures of Jurkat cell lines (Figure 1).

### Impact of the −237 SNP on mRNA and Soluble TNFα Production in BCLs

**Figure 2:**
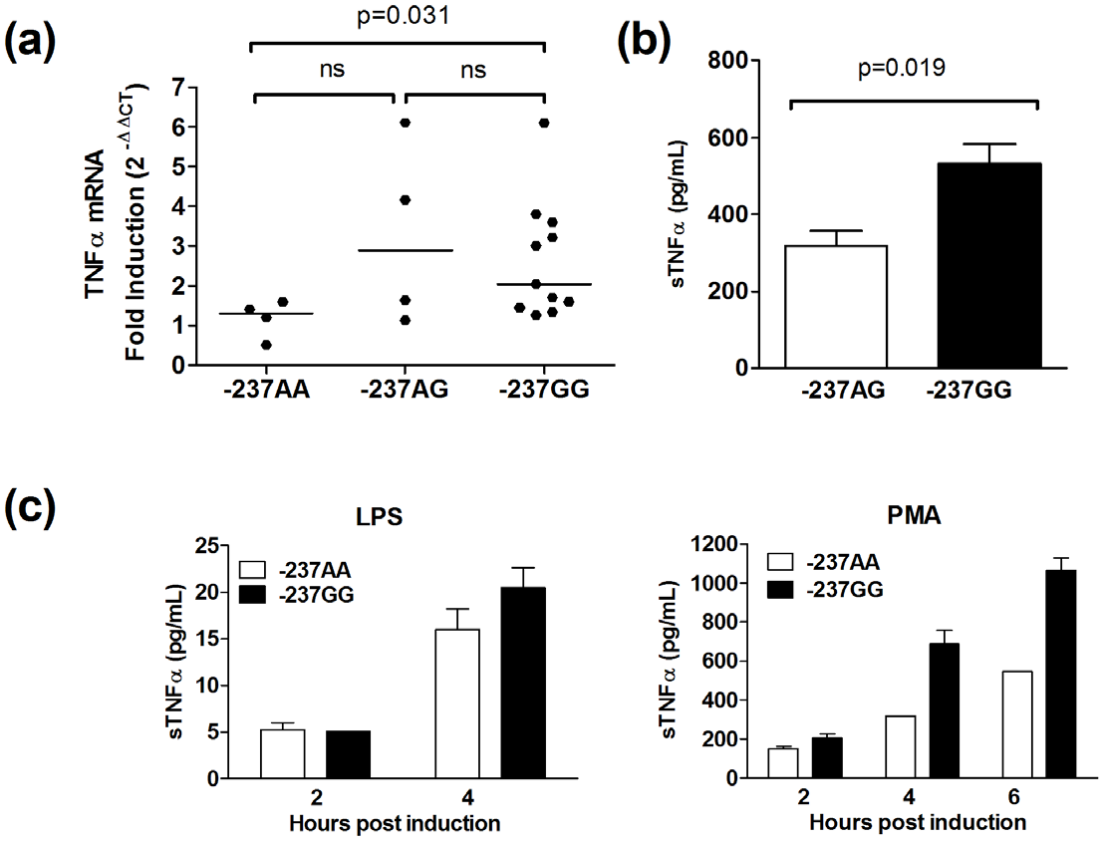
−237A homozygosity associates with reduced TNFα production in PMA-activated B cell lines. (a) Immortalised BCL lines generated from healthy donors who were −237 AA homozygous (n = 4), −237GG homozygous (n = 11) or GA heterozygous (n = 4) were stimulated for 4 hours with PMA, or left untreated, following which mRNA was isolated for qPCR analysis. TNFα mRNA fold induction (stimulated/non-stimulated) is reported. (b) One million BCLs from −237GG homozygous (n = 11) and GA heterozygous (n = 4) donors were stimulated with PMA for 4 hours, following which soluble TNFα (sTNFα) levels were measured by ELISA. The data is presented as absolute differences in sTNFα secretion in the −237 homozygous versus heterozygous group. (c) Stimulation of TNFα production on a −237AA background was reduced relative to −237GG in the presence of different stimuli. The data is presented as absolute differences in soluble TNFα secretion (pg/ml) for a single −237GG and −237AA homozygous BCL line. Wilcoxon-Mann-Whitney tests were used for statistical comparisons, and two-tailed P values are indicated in (a) and (b).

We then asked if the functional differences between the A and G −237 SNP variant promoters were evident under physiological settings. To do this we evaluated TNFα production in cell lines that carried these polymorphisms. Newly generated and low-passage immortalised BCLs from −237A/G heterozygous, and −237AA/GG homozygous donors were stimulated with PMA for 4 hours prior to mRNA isolation and quantitative PCR evaluation. TNFα mRNA expression levels were measured relative to GAPDH expression, which was quantified using inventoried Taqman primers and probes. A trend was observed which illustrated, in line with the luciferase promoter assays, that TNFα mRNA fold increase was less in individuals who carried the −237A SNP following stimulation, with a reduction in median TNFα transcript levels from −237GG to −237AA on a homozygous background. The differences between these two groups were significant. However, median TNFα fold induction was highest in −237AG heterozygous donors (Figure 2a).

Production of soluble TNFα was measured in follow-up analyses using a subset of the BCL lines tested for TNFα mRNA expression. Assay supernatants were harvested following 4 hours stimulation with PMA and soluble TNFα was measured by ELISA. As observed for TNFα mRNA expression, increased soluble TNFα (sTNFα) production was observed on a homozygous −237GG background, however, under these experimental conditions, was higher relative to both GA and AA donors (Figure 2b).

Collectively, our BCL-derived data suggested that promoters encoding the −237A variant generally demonstrated weaker activity relative to their common G variant counterparts. As all our assays were performed with a single concentration of PMA and for a fixed period of incubation we carried out a small-scale experiment to determine if these disparities were apparent under different experimental conditions. Our results illustrated that for BCLs, the intrinsic differences between these promoters were also evident when these variables were taken into account (Figure 2C).

### Impact of the −237 SNP Variant on TNFα Production in Peripheral Blood-derived Monocytes

**Figure 3:**
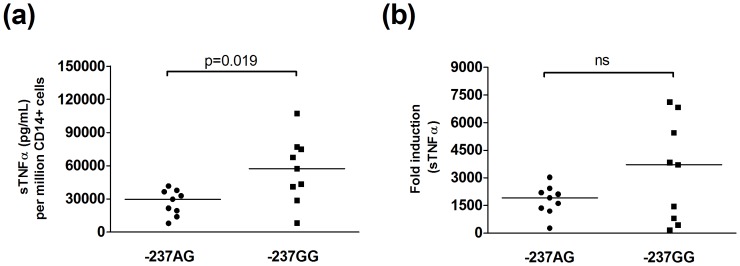
Reduced sTNFα production on a−237A SNP background following LPS-activation of PBMCs. 1 million PBMCs isolated from healthy −237GG (n = 9) homozygous or GA heterozygous (n = 9) donors were either left untreated, or stimulated for 4 hours with LPS, following which TNFα levels were estimated by ELISA. sTNFα datasets were evaluated relative to the number of CD14 positive monocytes present in each sample, and TNFα levels were adjusted to represent TNFα production per 1million CD14+ cells. (a) Absolute sTNFα production (stimulated minus non-stimulated) and (b) fold induction (stimulated/non-stimulated) were compared. Wilcoxon-Mann-Whitney tests were used for statistical comparisons, and two-tailed P values are indicated.

TNFα production is both stimulus and cell-type specific, hence we extended the study to evaluate monocytes which represent major producers of this cytokine *in vivo*. Using isolated PBMCs from healthy individuals who carried either the −237A (GA heterozygous) and −237G SNPs (GG homozygous) as a source of monocytes, we evaluated soluble TNFα production upon LPS stimulation. When soluble TNFα levels were evaluated per fixed number of PBMC input (1 million cells), there were no statistical differences between carriage of either SNP and absolute levels of TNFα production. However, when the datasets were corrected for the total number of CD14 positive cells per assay, and when sTNFα levels were adjusted and normalised relative to a fixed number of CD14 positive, CD3 negative cells then carriage of the −237A SNP on a heterozygous background was associated with lower TNFα production relative to −237G homozygosity, both in terms of TNFα production (subtraction of basal TNFα levels in absence of stimulation) and in relation to fold induction (stimulated/basal) (Figure 3).

### Transcription Factor (Tf) Binding Assays and Software Prediction Data

The −237 SNP is located in close proximity to the 200 bp core promoter, within a region corresponding to a putative Y box repressor site identified in mice. We questioned if the functional disparities between promoters containing the A and G variants could be explained by differences in the binding of Tfs, co-activators or repressor proteins in this region. Complementary oligos up to 30 base pairs in length and encoding either the A or G −237 polymorphisms were annealed and tested for binding to nuclear extracts purified from Jurkat cells by radioactive EMSA (data not shown). Despite various attempts using different annealing conditions, buffers, nuclear extracts, binding conditions, and experimental approaches, we did not detect binding of nuclear proteins to oligos contain either the G or A SNP variants.

We then utilised various Tf Binding prediction software programs to gauge whether Tf or repressors were predicted to bind the region encompassing the −237 SNP. Of the factors predicted, a proportion were not affected by the nature of the encoded −237 SNP variant, whereas others demonstrated exclusivity depending on the presence of either the G or A polymorphism (Table S1).

### Epigenetic Modifications Encompassing the −237 SNP

**Figure 4:**
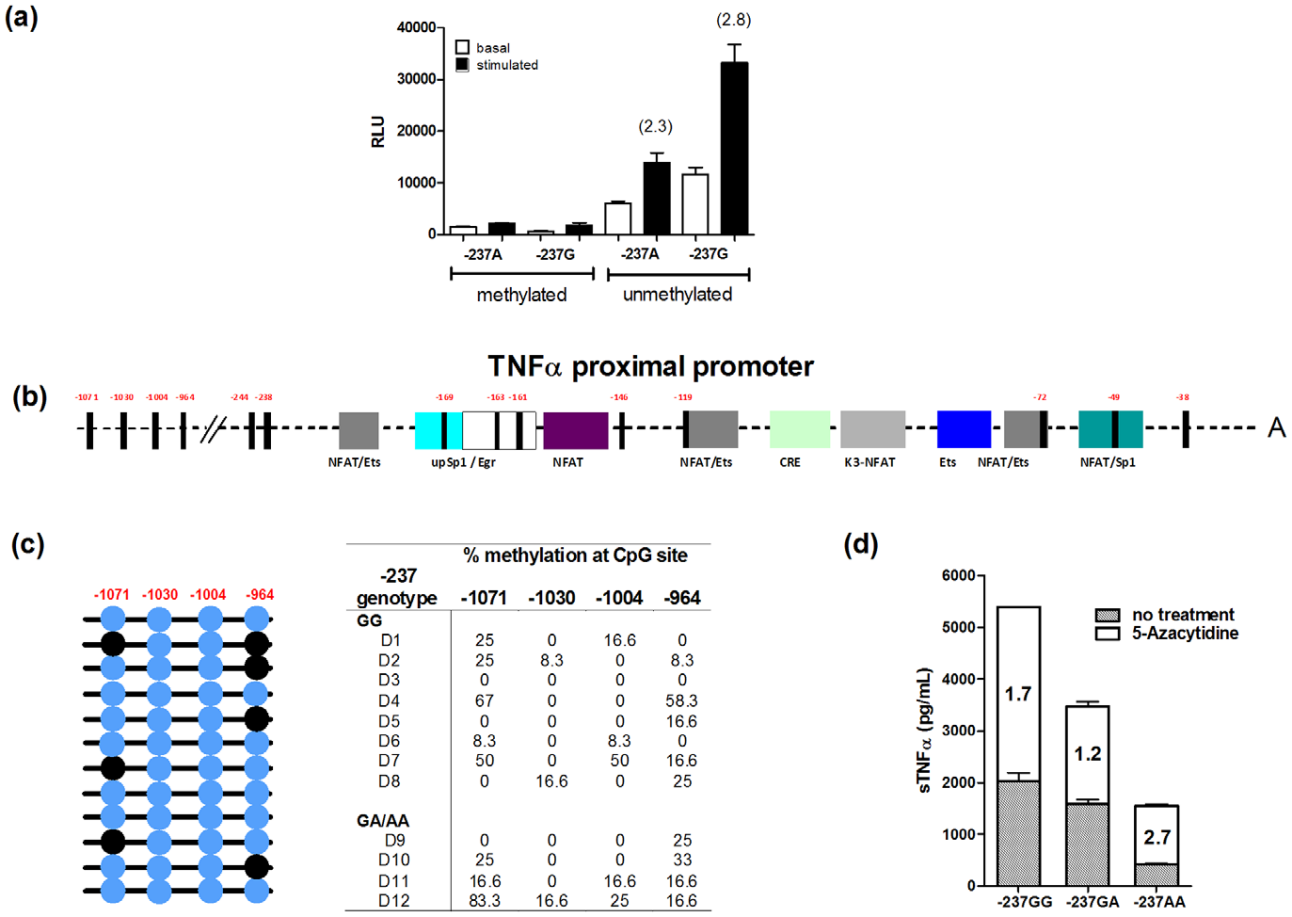
DNA methylation abrogates the activity of both SNP variant TNFα promoters but does not readily explain their functional differences. (a) Reporter luciferase genes under the control of TNFα promoters carrying either the −237A or G variant were methylated, or demethylated prior to transfection into Jurkat cells. Following 24 hours the cells were stimulated with PMA (for a further 4 hours) prior to the evaluation of luciferase activity. Fold changes (stimulated/non-stimulated) in TNFα production for unmethylated promoters are noted in parentheses (b) Outline of the proximal TNFα promoter adapted from previous reports [10], [43], illustrating the various transcription factors known to bind in this region. CpG dinucleotides including those which encompass the −237 and −1030 SNPs are noted as black strips, and their positioning relative to the Transcription Start Site (TSS) is displayed numerically (in red). (c) DNA isolated from −237 AA homozygous, −237GG homozygous and GA heterozygous BCLs was bisulfite converted, PCR amplified and sequenced to assess the methylation status of TNFα promoter sequences. A representative sequencing screen encompassing the −1030 SNP denotes the frequency of methylated cytidines (noted as black circles) within individual templates sequenced for a single donor, with a tabular version of summary data for a selection of BCL lines denoting the percentage of methylation at each CpG dinucleotide motif. (d) Three BCL lines encoding different combinations of the TNFα promoter −237 SNP variants (GG, GA and AA) were either left untreated or pre-incubated for 48 hours with 5-Azacytidine prior to PMA stimulation for 4 hours. sTNFα was subsequently measured by ELISA, and both absolute (bar chart) and sTNFα fold increase compared to their respective non-drug treated, PMA stimulated backgrounds (embedded values) are reported. Two biological and technical replicates were analysed per experiment, and median TNFα production plus SEM is reported.

The −237G to A mutation disrupts a CpG dinucleotide motif which is susceptible to DNA methylation *in vivo*; this is in close proximity to neighbouring CpG dinucleotide motifs near the 5′region of the core promoter. We argued that because the −237A variant alters the spacing and frequency of CpG dinucleotide motifs in this region it could impact the extent or pattern of local CpG methylation, and in turn TNFα production. Firstly, we evaluated the influence of CpG methylation on the activity of the individual TNFα promoters using both the −237G and −237A luciferase reporter plasmids. The plasmids were methylated prior to transfection, and transfected cells were subjected to a DNA methyltransferase inhibitor, 5-Azacytidine to prevent subsequent plasmid methylation upon incubation. Using this strategy we observed that methylation of both the −237A and G containing promoters severely disrupts luciferase activity, reinforcing the role played by DNA methylation in the activity of the proximal TNFα promoter (Figure 4a).

We next evaluated the DNA methylation status of the TNFα promoter in BCL lines to determine if carriage of either SNP affected the degree of CpG methylation in proximity to the core promoter. Our data, which was generated from PCR amplification, TOPO cloning and sequence analyses of bisulfite-converted DNA, suggested that the functional differences between these promoters were not readily explained by disparities in neither the extent nor the pattern of CpG in this region, and hence, the inherent functional differences between the −237 SNP variant promoters (data not shown). We extended screening to include mutations that encompass the TNFα promoter −1030 SNP (rs1799964), as this T to C variant is frequently inherited as a complotype with the −237A SNP and generates a methylation-susceptible CpG dinucleotide motif. Although the extent of methylation was greater in this region of the promoter, a correlation between TNFα production and the degree of methylation was not observed (Figure 4b-c).

Analysis of bisulfite-converted DNA through PCR amplification and TOPO cloning is limited in terms of in-depth sequence coverage, and data biases can also occur during the amplification and cloning of bisulfite-converted PCR products [19]. Therefore, to corroborate our methylation data obtained from bisulfite analyses, we performed cellular assays using the methylation inhibitor 5-Azacytidine, and compared soluble TNFα production on different −237 SNP backgrounds (AA/GG homozygous and GA heterozygous) using BCL lines either untreated or pre-treated for 48 hours prior to PMA stimulation. Although 5-Azacytidine pre-treatment led to elevated TNFα production in all BCL lines irrespective of their −237 genotype, the relative and absolute changes in TNFα production did not support an obvious role for DNA methylation as the major contributor to the −237 SNP-related differences in promoter activity (Figure 4d).

### TNFα Promoter Haplotypes in HIV Infected Individuals

HLA-B*5701 is associated with slower disease progression and although a large percentage of B*5701 positive individuals progress slowly to AIDS, a number of patients who carry this allele progress at normal rates. This is assumed to reflect differences in the interplay between CD8 and NK cell-mediated immune control, and the evolution of viral escape variants [20], [21]. We also questioned if specific differences existed in the TNFα promoters carried by HLA-B*5701 long term non-progressor (LTNPs) versus normal progressors (NPs), as these presumably could act additionally to influence disease dynamics. For a comprehensive screen of TNFα promoter sequences, we also included a number of normal progressors and LTNPs that were HLA-B*5701 negative. Analysis of the King’s College and John Warin Ward Clinical Cohort samples unveiled seven TNFα promoter haplotypes in which the clustering of the five most common TNFα SNPs appeared to be non-random (Table 1). Interestingly, the TNFα −237A variant segregated exclusively with the distant TNFα promoter-1030C SNP, and this combined genotype was observed in HLA-B*5701 positive patients. However, the −1030C variant was not exclusively carried on a HLA-B*5701 background and was present in haplotypes that carried the −237G SNP. Patients were also screened for carriage of the TNFα promoter −375A SNP (rs1800750) as previous studies in both African and European populations have reported linkage of this SNP with the −237A allele [13], [22]. However, in our samples of Caucasian origin, the −375 position was monomorphic for the common G SNP. As our patient cohorts included only two HLA-B*5701 patients classified as NPs we were unable to perform extensive sequence comparisons, however, based on the limited sequence datasets we observed strong concordance in sequence identity between promoters encompassing the −1030 to −237 region in NP and LTNPs groups.

**Table 1:** Observed TNFα promoter haplotypes and frequencies in HIV-1 infected patient groups.

Observed TNFα Haplotypes					Haplotypic frequency number (%)		
−1030	−862	−856	−307	−237	LTNPs (n = 60)	NPs (n = 30)	P
T	C	C	G	G	24 (40)	20 (64.5)	**0.025**
T	C	C	A	G	6 (10)	3 (9.7)	1.00
T	C	T	G	G	3 (5)	0	0.548
C	C	C	G	A	13 (21.7)	2 (6.5)	0.082
C	A	C	G	G	12 (20)	2 (6.5)	0.129
C	C	C	G	G	1 (1.7)	3 (9.7)	0.106
**T**	A	C	G	G	1 (1.7)	0	1.000

Five of the common Caucasian ancestral TNFα promoter SNPs were typed by sequencing and confirmed by RFLP analysis. Statistical analysis was based on the total number of haplotypes (2n) within each group. P values were calculated using the Fisher’s exact test. Statistically significant values are shown in bold. The −237A promoter haplotype was exclusively observed in patients who carried HLA-B*5701.

## Discussion

The major impact of HLA, mostly notably MHC class I subtypes, on clinical outcome in HIV-1 infection has recently been re-iterated in large GWAS studies. Yet because of the complexities surrounding these approaches, specifically factors relating to cohort selection and linkage disequilibrium (LD), it has proved difficult to identify independent causal variants associated with favourable immune control [3], [4], [5], [6], [7]. This is best exemplified by a number of the candidate SNPs identified within the HLA region on Chromosome 6, whose proposed disease-influencing effects can be now explained by their association with ‘protective’ MHC class I alleles. However, additional SNPs within this region could play an auxiliary role influencing disease dynamics, but remain overlooked owing to extensive linkage in this region, and due to limitations in the approaches currently employed to perform such analyses [23]. In this study we focused on the ancestral TNFα promoter −237A variant (rs361525), which is in strong linkage disequilibrium with the ‘protective’ HLA-B*5701 allele [8], [24]. Although many of the ancestral SNPs within the extended TNFα promoter have been associated with a variety of autoimmune conditions and disease outcomes, data pertaining to the direct role of these variants, including the −237 SNP, on TNFα expression remains controversial [25]. However, in light of its strong linkage with HLA-B*5701, and given the proximity of the −237 SNP to the core promoter, within both a putative repressor region (characterized in mice) and a methylation-susceptible CpG dinucleotide motif, we chose to re-appraise its putative impact on TNFα production. Using a variety of cell lines, stimuli and experimental read-outs we consistently detected lower TNFα production on a −237A variant background, a finding observed at the level of both TNFα transcription and translation. It is unlikely that our data was confounded by factors relating to post-transcriptional modifications, such as tristetraprolin or miRNA-mediated down-regulation, for example, as the functional impact of this SNP was also observed in a luciferase assay reporter system lacking canonical TNFα 3′UTR regions.

The −237 SNP represents one of ten common TNFα promoter SNPs linked to ancient human HLA haplotypes that denote evolutionary markers of ancestry traceable to primate lineages [8], [26]. Although an uncommon Caucasians variant, this SNP is observed at higher frequencies in Asian, Amerindians and African populations. In our healthy volunteers and HIV-1 patients of Caucasian origin, the −237A SNP was linked to the HLA-B*5701 and B*18 haplotypes as described previously [27]. In HLA-B*5701 individuals, this genotype was observed in conjunction with the rarer −1030C SNP, and represents a TNFα promoter complotype also identified in other ethnic groups, and in the absence MHC class I-defining haplotypes [8]. In addition to its emergence in the *Sympahalangus* genera of small apes, the −237A/−1030C complotype evolved independently in humans, and based on population frequencies is likely to have originated in Africa prior to migration into Asia and Europe [28]. Presumably, co-evolution of the −1030C/−237A complotype was selected due to their linkage with additional genes in the MHC region. Alternatively, factors relating sequence limitations such as genomic stability or DNA accessibility could also have driven their combined evolution *in vivo*.

As the −1030 SNP introduces a CpG dinucleotide motif and the −237A SNP disrupts an existing site this complotype could presumably impact the promoter epigenetically via processes relating to DNA methylation. DNA methylation is known to play an important role in the function of the human TNFα promoter, a finding re-capitulated in our plasmid-based 5-Azacytidine experiments [29]. Significant tissue-specific inter-individual variation in the methylation of the TNFα 5′UTR region has been described in humans [30], which could result due to the lower CpG density and lack of canonical CpG island in the TNFα promoter [31]. There is also strong evidence supporting the impact of genotype on the epi-genotype, and this is often directly related to the presence of CpG sites within with inherited promoter SNPs [32]. Hence, differential DNA methylation could represent a key feature impacting TNFα promoter activity in different tissue and presumably cell types *in vivo*. When we explored the methylation status surrounding the −237 SNP in BCLs we did not observe overt differences between the methylation profiles in terms of the extent, pattern and frequency of promoters carrying either the A or G SNP, and CpG methylation was low for all promoters in this region. Although we did detect inter-individual differences in the methylation status of the distal promoters in the region encompassing the −1030 SNP, a clear relationship between the degree or pattern of DNA methylation and promoter activity, and the presence of either SNP in this region was not apparent.

In summary, we observed that the carriage of the −237A SNP in the human TNFα promoter is associated with a modest reduction in TNFα production, a finding that appears independent of the cell-types and stimuli tested. Although the number of subjects in this study was low, the variety of different approaches illustrated a common result. In the setting of HIV-1 infection, the A variant SNP could represent an independent contributory SNP affecting the course of disease progression, although its impact is presumably minor relative to that conferred by the B*5701 allele, particularly as the A SNP is also inherited with MHC class I types not associated with favourable immune control. Nonetheless, a modest impact on TNFα production may be significant, as positive feedback loops leading to enhanced TNFα production might amplify the impact of this SNP *in vivo* [33]. This is especially important during HIV-1 infection, where minor differences in TNFα production could (i) directly affect HIV-1 replication, as TNFα is known to activate HIV-1 replication through activation of its LTRs, consistent with a positive correlation between plasma TNFα levels and HIV plasma load [34], [35], [36], [37] (ii) influence the spread and frequency of HIV-1 ×4 and R5 viruses, through the direct impact of TNFα on the expression of the HIV-1 co-receptors CXCR4 and CCR5, on permissive cell types [38] and (iii) affect the degree of hyper-immune activation following translocation of potent TNFα-inducing stimuli, including LPS, into the systemic circulation following HIV-1-associated destruction of gut mucosal integrity [39]. The mechanism by which the −237A SNP affects TNFα production is not related to the differential methylation of the SNP-variant TNFα promoters. Nor are the differences explained by the specific binding of nuclear factors, and our data is consistent with previous findings illustrating DNAse I hypersensitivity in this region of the TNFα promoter [13]. However, despite our inability to detect Tf or repressors using traditional EMSA-based approaches, we cannot exclude the possibility that specific factors were responsible for the disparities between the SNP variant promoters, particularly in light of prediction software data. Presumably, the intrinsic differences between the −237 SNP variant promoters could relate to genomic modifications due to DNA accessibility or higher-order chromatin structure, which could in turn affect the kinetics and/or composition of the TNFα enhanceosome. Higher throughput approaches are now underway to more accurately assess these possibilities.

## Materials and Methods

### Patient Enrolment

HIV-1 infected subjects were enrolled from both the King’s College long term non-progressor (LTNP) Cohort and the John Warin Ward in Oxford for the evaluation of TNFα promoter haplotypes and SNP analyses. Ethical approval was obtained from the relevant Ethical Committees (Local Research Ethics Committee (LREC) at King’s College, London ((LREC 99-075) and the NHS Research Ethical Committee (formerly Central Office for Research Ethics Committees (COREC 96.108)) and all patients gave written consent to donate blood. As healthy control subjects were independently and anonymously recruited for typing and cellular studies and because all staff working on this project were fully-blinded to their identity, ethical consent was not required. Nonetheless, all donors were fully informed and gave verbal consent to donate a one-off sample of blood for the purpose of this investigation. HLA class I typing was performed by the amplification refractory mutation systems PCR (ARMS-PCR) using sequence specific primers.

### Cells and Cell Lines

Peripheral blood mononuclear cells (PBMCs) were isolated from heparinised venous blood by Ficoll-Hypaque (Nycomed, Oslo, Norway) density gradient centrifugation, and were subsequently washed in RMPI 1640 (Sigma-Aldrich, Dorset, UK) supplemented with 10% foetal calf serum, 2 mM Glutamine 100 IU of penicillin/L, 100 mg of streptomycin/L (referred to herein as R10) (Gibco-BRL, Groningen, the Netherlands). PBMCs were cryopreserved in 10%DMSO/foetal calf serum for subsequent analyses.

The Jurkat E6.1 cell line (purchased from ECCAC) and EBV-immortalised B cell lines (BCLs) were maintained in R10 at 37°C in a 5% CO_2_/95% air incubator. EBV-immortalised BCLs were generated in-house according to previously published methods [40].

### DNA Extraction and TNFα Promoter Amplification

Genomic DNA was extracted from cryopreserved PBMCs using the Qiagen Gentra Puregene DNA extraction kit (Qiagen, West Sussex, UK). Approximately 1.35 kb of the TNFα promoter was amplified by PCR using primers; Fw2 (5′-GCA GGG AGG GGA CTA TTT ATG AAG G-3′) and Rv1 (5′-GGG ATT TGG AAA GTT GGG GAC AC-3′). The resulting PCR products were cloned into pCR-4 TOPO TA cloning vectors (Invitrogen) and sequenced. Results obtained from sequence analyses were also verified by Restriction Fragment Length Polymorphism (RFLP) analysis using previously published methodologies [41], [42].

### Generation of the -237A Reporter Plasmid

The TNFα Translucent Gene Promoter Reporter Vector, which naturally encodes the -237 G variant, was obtained from Panomics. The −237A mutation was introduced into this vector using QuikChange® II XL Site Directed Mutagenesis Kit (Stratagene, La Jolla, USA) according to the manufacturer’s instructions. In brief, two mutagenesis oligonucleotides (5′-AAG ACC CCC CTC GGA ATC AGA GCA GGG AGG ATG-3′ and 5′-CAT CCT CCC TGC TCT GAT TCC GAG GGG GGT CTT-3′) were used to PCR amplify the TNFα Translucent Gene Promoter Reporter Vector. Template DNA was subsequently digested using the DNA methylated specific endonuclease *DpnI,* leaving the mutated variant vector, which was transformed into *Escherichia coli* strain XL10 Gold and recovered using the EndoFree Plasmid Maxi Kit (Qiagen, West Sussex, UK). The presence of the −237A mutation was verified by sequencing.

### Transfections and Luciferase Assay

Transfection of Jurkat cells with the TNFα Translucent Gene Promoter Reporter Vectors carrying both the −237G and A variants was carried out using Lipofectamine LTX and PLUS Reagent (Invitrogen, Carlsbad, USA). The day before transfection, cells were split to a density of 5×10^5^ to 1×10^6^ cells/ml. For transfections, 1.5 µg of plasmid DNA was diluted in 100 µl of OptiMem media, 1.5 ul of PLUS reagent and 3.75 ul of Lipofectamine LTX, in the order and timelines recommended by the manufacturer. 100 µl of the DNA/PLUS/Lipofectamine LTX complex was added to 5×10^5^ cells in 0.5 ml of complete growth media and incubated overnight at 37°C in a 5% CO_2_/95% air incubator. Cells were then stimulated with either 1 µg/ml ionomycin and 50 ng/ml PMA diluted in DMSO, in DMSO only, or left untreated and incubated at 37°C in a 5% CO_2_/95% air incubator for a further 6 hours. Following incubation, the cells were harvested and re-plated at a density of 6×10^4^ cells per well in a U–bottom 96 well plate. Following centrifugation, the media was carefully removed by aspiration, and the cells pellets were lysed with 20ul of Lysis Buffer (Promega, Luciferase Assay System) in conjunction with a freeze-thaw cycle. 100µl of Luciferase Assay Substrate Buffer was then added (Promega, Luciferase Assay System), and Luciferase activity was measured immediately on a Fusion 3.5 Universal Microplate Analyzer (Perkin-Elmer, UK).

### qPCR and ELISA-based TNFα Evaluation

Newly established and low passage BCLs from healthy −237AG heterozygous and −237GG/AA homozygous donors were stimulated with PMA (50 ng/ml) for 4 hours prior to RNA extraction (kit) and cDNA synthesis (kit). TNFα primers and probes, sourced from Applied Biosystems (TaqMan Gene Expression Assay for TNF, inventoried stock Hs99999043_m1), were used to amplify cDNA. Results were normalised relative to GAPDH expression, using inventoried human GAPDH endogenous control primers and FAM/MGB probes (Applied Biosystems). The final datasets were normalised for relative expression, using the 2^−ΔΔCT^ method.

Following PMA stimulation of BCL targets for 4 hours, assay supernatants were harvested and TNFα was quantified using the Human TNFα ELISA Ready-Set-Go kit (eBioscience).

### 5-Azacytidine Experiments

Luciferase reporter plasmids carrying the −237A and G SNP were methylated prior to transfection using CpG Methyltransferase (M.SSS I). Methylation was confirmed by digestion with the CpG methylation-sensitive enzyme, BstUI, and agarose gel electrophoresis. A fraction of transfected Jurkat cells were incubated with 500 nM of the DNA methyltransferase inhibitor 5-Azacytidine to prevent subsequent plasmid methylation upon transfection. Transfection and luciferase assay protocols were performed according to those described in the Transfections and Luciferase Assay section outlined above.

BCLs encoding the GG, AA and GA SNPs at −237 were either pre-treated with 500 nM 5-Azacytidine or left untreated for 2 days. Cells were subsequently harvested, plated at 1mg/ml and stimulated with 50 ng/ml of PMA for 4 hours, following which assay supernatants were harvested. sTNFα was assayed using a commercially available Human TNFα ELISA Ready-Set-Go kit (eBioscience).

### Bisulfite PCR

Genomic DNA was extracted from the different cell types using PureGene DNA purification kit (Gentra System, Minneapolis, USA) and 500 ng of genomic DNA was bisulfite converted using Methylcode™ Bisulfite Conversion Kit (Invitrogen, Corp., Carlsbad, USA) according to manufacturer’s instructions. PCR-generated fragments were amplified using the following primers: −237 amplification: 5′-TTT GTA TTT TGT TTG GAA GTT AGA AGG AAA TAG ATT A-3′ and 5′-AAA ACT TCC TTA ATA AAA AAA CCC ATA AAC TCA TC-3′; −1030 amplification; 5′-AGA GTT GTG GGG AGA ATA AAA GGA TAA GG-3′ and 5′-AAA CAT TCT CCT ACC CAT TAC TAT AAT CAC ATC TC -3′. PCR products were subcloned into the pCR4 TOPO TA cloning vector (Invitrogen, Corp., Carlsbad, USA) and sequenced.

### Electrophoretic Mobility Shift Assay (EMSA)

5′-ACGT-3′ overhangs on annealed oligonucleotides containing the −237G and A SNPs were radioactively labeled with 32P, and incubated with 2 µg nuclear extract (previously prepared from Jurkat cell) for 10 minutes in a reaction containing standard Hepes buffer, pH 7.8. Reactions were resolved on a 4.5% polyacrylamide gel (200 V for 150 minutes at 4°C). Vacuum dried gels were exposed to hyperfilm (Amersham).

## Supporting Information

Table S1 Transcription factor binding prediction analyses for the −237A and −237G promoter variants. TNFα promoter sequences encoding the −237A and −237G SNP variants were evaluated using PROMO and MatInspector Software. Factors predicted to bind exclusively to DNA coding either the A or G variants are reported.
(DOC)
