# Supplementary material for: Functional Differences Exist between TNFα Promoters Encoding the Common −237G SNP and the Rarer HLA-B*5701-Linked A Variant
Source: PLoS One. 2012 Jul 13;7(7):e40100. doi: 10.1371/journal.pone.0040100 (PMC3396654; doi:10.1371/journal.pone.0040100)
Supplement: Table S1 — Transcription factor binding prediction analyses for the −237A and −237G promoter variants. TNFα promoter sequences encoding the −237A and −237G SNP variants were evaluated using PROMO and MatInspector Software. Factors predicted to bind exclusively to DNA coding either the A or G variants are reported. (DOC) [file pone.0040100.s001.doc]

**Table S1. Transcription factor binding prediction analyses for the -237A and -237G promoter variants**

| **Prediction Program** | **-237G specific** | **-237A specific** |
| --- | --- | --- |
| PROMO | p300  BTEB3  Elk-1 |  |
| MatInspector | AP2  CDPCD3  GABP  VDR | GATA  GFI1  HAND1E47  MAF  OCT1  PAX5  PAX8  PARA |

TNFα promoter sequences encoding the -237A and -237G SNP variants were evaluated using PROMO and MatInspector Software. Factors predicted to bind exclusively to DNA coding either the A or G variants are reported.
